# Association of weight range with telomere length: A retrospective cohort study

**DOI:** 10.3389/fendo.2023.1106283

**Published:** 2023-04-11

**Authors:** Xinyu Wang, Jingli Wen, Qiang Qu, Shujun Gu, Lixi Zhang, Yu Li, Xu Qi

**Affiliations:** ^1^ Department of Respiratory and Critical Care Medicine, The First Affiliated Hospital of Nanjing Medical University, Nanjing, China; ^2^ Department of Cardiology, The First Affiliated Hospital of Nanjing Medical University, Nanjing, China; ^3^ School of Medicine & Holistic Integrative Medicine, Nanjing University of Chinese Medicine, Nanjing, China; ^4^ Department of Respiratory and Critical Care Medicine, The Affiliated Jiangsu Shengze Hospital of Nanjing Medical University, Suzhou, China

**Keywords:** weight range, telomere length, aging, metabolic dysregulation, obesity, NHANES

## Abstract

**Objective:**

Previous research has shown a significant association between weight and telomere length, but did not take into consideration weight range. The study was to investigate the association of weight range with telomere length.

**Methods:**

Data of 2918 eligible participants aged 25-84 years from the National Health and Nutrition Examination Survey (NHANES) 1999-2000 cycle were analyzed. Information about demographic variables, lifestyle factors, anthropometric variables, and medical comorbidities were included. Univariate and multivariate linear regression model with adjustments for potential confounders were employed to determine the association between weight range and telomere length. A non-parametrically restricted cubic spline model was used to illustrate the possible non-linear relationship.

**Results:**

In univariate linear regression, BMI_max_, BMI range, and weight range all revealed significant negative associations with telomere length. However, annual rate of BMI/weight range showed a significant positive associations with telomere length. There was no significant association between telomere length and BMI_min_. After adjusting for potential confounders, the inverse associations persisted in BMI_max_ (β=-0.003, P<0.001), BMI range (β=-0.002, P=0.003), and weight range (β=-0.001, P=0.001). Furthermore, annual rate of BMI range (β=-0.026, P=0.009) and weight range (β=-0.010, P=0.007) presented negative associations with telomere length, after adjusting for covariates in Model 2-4. The association between BMI_min_ (β =-0.002, P=0.237) and telomere length still could not reach statistical significance in multivariate linear regression model. The results of restricted cubic spline analysis showed that BMI_max_ (P for nonlinear =0.026), BMI range (P for nonlinear =0.022), weight range (P for nonlinear =0.035), annual rate of BMI range (P for nonlinear =0.030), and annual rate of weight range (P for nonlinear =0.027) all had nonlinear inverse associations with telomere length.

**Conclusions:**

The study suggests that weight range is inversely associated with telomere length in U.S. adults. Larger weight fluctuation may accelerate telomere shortening and aging.

## 1 Introduction

Telomeres are the protective repeats of nucleotides at the ends of chromosomes, which can shorten with normal cell division and finally trigger cellular senescence (1). Studies have revealed that chronic inflammatory states, oxidative stress, as well as unhealthy lifestyle and nutritional status can lead to telomere attrition through oxidative DNA damage in telomeric region and telomerase dysfunction (2). Moreover, evidence suggests that accelerated telomere shortening is associated with adverse health conditions, including infection (3–5), cardiovascular disease (6), cancer (7), and mental illness (8).

Obesity, a global public health problem, has been increasing in a rapid manner to date (9). It has been reported in a prospective cohort study that a stable obesity status throughout adulthood, and weight gain from young adulthood to middle adulthood, were associated with higher mortality risks (10). Another population-based cohort study has discovered a j-shaped relationship between death causes (including cancer, heart disease, and respiratory diseases) and body mass index (BMI), with the lowest risk seen in the range 21 to 25 kg/m^2^ (11). On a molecular scale, both obesity and ageing facilitate mitochondrial dysfunction, inflammation, and cellular senescence via redox imbalance and insufficient autophagy (12).

Accumulating studies have showed that weight might be related to telomere length. Valdes et al. (13) has emphasized obesity had the effect of accelerating telomere erosion and contributing to aging. Several studies have revealed that obesity-induced inflammation mediates the inverse association of BMI with telomere length to some extent (12, 14). According to a cross-sectional study of 3886 participants, a 10-year weight gain was associated with telomere shortening and promoted aging across adulthood (15). However, previous studies did not consider the association of weight range with telomere shortening during the stages of adulthood. The purpose of this study was to examine the association of weight range with telomere length, including the maximum, minimum, range, and annual rate of change, with the help of the National Health and Nutrition Examination Survey (NHANES) database.

## 2 Materials and methods

### 2.1 Study population

All data were publicly available from the NHANES 1999-2000 cycle. NHANES, a nationwide ongoing cross-sectional study in the United States, collected health and nutrition information among the noninstitutionalized population through interviews, physical examination and laboratory testing. More detailed information about study design and data collection of NHANES are published on the official website (16).

The NHANES 1999-2000 cycle had a total of 9965 subjects participated in. Considering cell division and metabolism occur exuberantly until the age of 25, the maximum of weight/BMI is more likely to appear after the age of 25 (17). All participants aged 85 years and older in NHANES were recorded as 85 years. Therefore, we included participants aged 25 to 84 years (n=4257) and excluded the participants who had missing data on telomere length (n=1108) or the indicators of weight range (n=697). We finally had 2918 eligible participants enrolled for analyses. National Center for Health Statistics Research Ethics Review Board has reviewed and approved the study, and written informed consent was obtained from all participants.

### 2.2 Indicators of weight range

The maximum and minimum of weight and the corresponding age were recalled at the NHANES questionnaires. Participants more than 18 years old were asked to recall the maximum lifetime weight and the exact age, and participants more than 19 years old were asked to recall the minimum weight since age 18 and the exact age. The maximum (BMI_max_, kg/m^2^) and minimum (BMI_min_, kg/m^2^) of BMI were calculated as the corresponding weight (kg) divided by the square of height (m^2^). We calculated the maximum minus the minimum, generating weight range (kg) and BMI range (kg/m^2^). In addition, we calculated the time between the maximum and minimum values. Annual rate of weight (kg/year) and BMI range (kg/m2/year) were calculated by dividing the corresponding range by the time. All calculations took into account the direction in which the change occurred.

### 2.3 Telomere length measurement

The details of the telomere length measurement were reported on the official website of NHANES (18, 19) and elsewhere previously (20). The peripheral blood samples were obtained from participants in the NHANES surveys. Measurement procedure started from the extraction and purification of deoxyribonucleic acid (DNA). Quantitative polymerase chain reaction (qPCR) was used to determine telomere length in the laboratory of Dr. Elizabeth Blackburn at the University of California, San Francisco. Telomere length was measured relative to standard reference DNA (T/S ratio) which came from the human diploid fibroblast cell line IMR90. Each sample was assayed on duplicate wells which were blinded to the investigators. Eight control DNA samples were set in each assay plate, which were applied to normalize between-run variability. Runs which had more than 4 control DNA values falling outside 2.5 standard deviations from the mean for all assay runs would be identified as potential outliers and excluded from further analysis (<6% of runs). Telomere length were presented as the T/S ratio finally.

### 2.4 Covariates

Covariates were constitutive of demographic variables, anthropometric variables, lifestyle factors, and medical comorbidities. Information about demographic variables [age, sex, ethnicity, marital status education, and family income-poverty ratio (PIR)], lifestyle factors (smoking status and alcohol use), and medical comorbidities [baseline history of diabetes, hypertension, cardiovascular disease (CVD), chronic obstructive pulmonary disease (COPD), and cancer] were collected by means of questionnaires. The anthropometric variables (baseline height and weight) were acquired via physical examination.

Among them, continuous variables included age (year), weight (kg), height (m), and BMI (kg/m^2^). Categorical variables included sex (male and female), ethnicity (non-Hispanic white, non-Hispanic black, Mexican American, and other), marital status (married/living with partner, widowed/divorced/separated, and never married), education (more than high school, less than high school, and high school), PIR (low income, medium income, and high income), baseline BMI (underweight, normal weight, overweight, and obesity), smoking status(never, former, and now), alcohol use (never, mild, moderate, heavy, and never), and baseline history of diabetes, hypertension, cardiovascular disease, chronic obstructive pulmonary disease, and cancer. Detailed explanation for covariate acquisition process is available on the official website of NHANES.

### 2.5 Statistical analysis

Participants with telomere length values and the indicators of weight range (BMI_max_, BMI_min_, weight range, BMI range, and annual rate of weight and BMI range) are retained. Outliers were handled by means of interquartile range (IQR), defined as values which were more than 1.5 times the IQR from the boundary of IQR (21). The lower outliers were replaced by the 25th percentile minus 1.5 times the IQR, and the higher were replaced by the 75th percentile plus 1.5 times the IQR. For missing of other covariates, we used multiple multivariate imputations to maximize statistical power and minimize bias caused by data excluded from analyses (22).

For continuous variables, we performed normality test by means of the Kolmogorov-Smirnov normality test. Mean ± standard deviation was used to describe normally distributed continuous variables, while median (Q1, Q3) to non-normally distributed continuous variables. Categorical variables were presented as frequency (n) and proportion (%). Among different telomere length groups (quartile), the one-way analysis of variance in normally distributed continuous variables, the Kruskal-Wallis test in non-normally distributed continuous variables and the chi-square test in categorical variables were performed to test the statistical differences. A boxplot was used to illustrate the distribution of telomere length according to the BMI range (deciles).

To investigate whether weight range are correlated with telomere length, univariate and multivariate linear regression models were employed. Four models were constructed to adjust for confounding effects of different covariates. Model 1: no covariates were adjusted. Model 2: adjusted for age, sex, and ethnicity. Model 3: adjusted for covariates in Model 2 plus educational level, family PIR, alcohol use, and smoking status. Model 4: adjusted for covariates in Model 3 plus medical comorbidities, including diabetes, hypertension, CVD, COPD, and cancer. Furthermore, we investigated the possible non-linear relationship between the weight range and telomere length by non-parametrically restricted cubic splines (23).

To test the robustness of the results, sensitivity analysis was performed by removing participants with any missing values and running the same statistical analysis. Subgroup analyses were performed through univariate and multivariate linear regression models in Model 1 and Model 4. We calculated the associations in different subgroups, including age (<60 or ≥60 years), sex (male or female), race/ethnicity (non-Hispanic white, non-Hispanic black, Mexican American, or other), smoking status (never, former, or now), alcohol use (never, mild, moderate, heavy, or never).

All data were statistically analyzed by R software (version 4.2.1). We considered that a two tailed P value of less than 0.05 to be statistically significant.

## 3 Result

### 3.1 Baseline characteristics of participants

Data of 2918 eligible participants were ultimately included in the study and analyzed statistically. The circumstantial flowchart was displayed in **Figure S1** (**Additional File 1**). The telomere length among the final participants ranged from 0.389 to 1.626 T/S ratio, with a median (Q1, Q3) of 0.960 (0.805, 1.134) T/S ratio. The summary of general characteristics categorized by telomere length groups (quartile) was provided in **Table 1**. According to the result of normality test, continuous variables in the study were non-normally distributed. Compared with those with longer telomere length, the participants with shorter telomere length tended to be older, male, Mexican American, and widowed/divorced/separated; besides, worse economic conditions, lower educational levels, and more comorbidities often accompanied them. According to the results of self-reported information, the median (Q1, Q3) age at least weight and greatest weight were 18 (18, 23) and 41 (30, 56) years. The median (Q1, Q3) of least weight and greatest weight were 58.97 (52.16, 68.04) and 81.65 (71.21, 96.16) kg respectively. An interesting finding, the inverse association between age at greatest weight and telomere length, exist in **Table 1**, but it did not remain after adjusting for the covariates in Models 2-4 (**Table S1**; **Additional File 1**).

**Table 1:** Baseline characteristics of study participants in the NHANES 1999-2000 cycle.

Characteristics	Overall	Telomere length (T/S ratio)				
		Q1[0.389,0.805]	Q2 (0.805,0.960]	Q3 (0.960,1.134]	Q4 (1.134,1.626]	*P*-value
Participants (n)	2918	727	735	727	729	
Age (years)	51.00 [37.00, 65.00]	62.00 [48.00, 73.00]	53.00 [39.00, 66.00]	46.00 [35.00, 62.00]	41.00 [32.00, 54.00]	<0.001
Sex (%)						
Male	1431 (49.0)	394 (54.2)	350 (47.6)	344 (47.3)	343 (47.1)	0.016
Female	1487 (51.0)	333 (45.8)	385 (52.4)	383 (52.7)	386 (52.9)	
Ethnicity (%)						<0.001
Non-Hispanic White	1405 (48.1)	329 (45.3)	381 (51.8)	365 (50.2)	330 (45.3)	
Mexican American	758 (26.0)	246 (33.8)	194 (26.4)	169 (23.2)	149 (20.4)	
Non-Hispanic Black	488 (16.7)	93 (12.8)	100 (13.6)	127 (17.5)	168 (23.0)	
Other	267 (9.2)	59 (8.1)	60 (8.2)	66 (9.1)	82 (11.2)	
Marital status (%)						<0.001
Married/living with partner	1986 (68.1)	490 (67.4)	513 (69.8)	491 (67.5)	492 (67.5)	
Widowed/divorced/separated	634 (21.7)	197 (27.1)	160 (21.8)	153 (21.0)	124 (17.0)	
Never married	298 (10.2)	40 (5.5)	62 (8.4)	83 (11.4)	113 (15.5)	
PIR level (%)						0.001
Low income	492 (16.9)	122 (16.8)	128 (17.4)	125 (17.2)	117 (16.0)	
Medium income	1251 (42.9)	360 (49.5)	303 (41.2)	285 (39.2)	303 (41.6)	
High income	1175 (40.3)	245 (33.7)	304 (41.4)	317 (43.6)	309 (42.4)	
Education level (%)						<0.001
Less than high school	1061 (36.4)	323 (44.4)	262 (35.6)	251 (34.5)	225 (30.9)	
High school	670 (23.0)	165 (22.7)	172 (23.4)	158 (21.7)	175 (24.0)	
More than high school	1187 (40.7)	239 (32.9)	301 (41.0)	318 (43.7)	329 (45.1)	
Height (meters)	1.67 [1.60, 1.74]	1.67 [1.59, 1.73]	1.66 [1.59, 1.74]	1.67 [1.60, 1.75]	1.67 [1.61, 1.74]	0.065
Body mass index at baseline (kg/m2)						0.07
Underweight	34 (1.2)	6 (0.8)	11 (1.5)	6 (0.8)	11 (1.5)	
Normal weight	836 (28.6)	178 (24.5)	201 (27.3)	229 (31.5)	228 (31.3)	
Overweight	1052 (36.1)	284 (39.1)	262 (35.6)	253 (34.8)	253 (34.7)	
Obesity	996 (34.1)	259 (35.6)	261 (35.5)	239 (32.9)	237 (32.5)	
Smoke (%)						<0.001
Never	1446 (49.6)	343 (47.2)	344 (46.8)	368 (50.6)	391 (53.6)	
Former	858 (29.4)	261 (35.9)	243 (33.1)	184 (25.3)	170 (23.3)	
Now	614 (21.0)	123 (16.9)	148 (20.1)	175 (24.1)	168 (23.0)	
Alcohol intake (%)						0.001
Never	388 (13.3)	105 (14.4)	90 (12.2)	86 (11.8)	107 (14.7)	
Former	605 (20.7)	177 (24.3)	163 (22.2)	141 (19.4)	124 (17.0)	
Mild	982 (33.7)	249 (34.3)	259 (35.2)	236 (32.5)	238 (32.6)	
Moderate	403 (13.8)	95 (13.1)	96 (13.1)	104 (14.3)	108 (14.8)	
Heavy	540 (18.5)	101 (13.9)	127 (17.3)	160 (22.0)	152 (20.9)	
Diabetes (%)	380 (13.0)	125 (17.2)	109 (14.8)	79 (10.9)	67 (9.2)	<0.001
Hypertension (%)	1372 (47.0)	424 (58.3)	364 (49.5)	338 (46.5)	246 (33.7)	<0.001
CVD (%)	322 (11.0)	132 (18.2)	94 (12.8)	58 (8.0)	38 (5.2)	<0.001
COPD (%)	74 (2.5)	29 (4.0)	25 (3.4)	10 (1.4)	10 (1.4)	0.001
Cancer (%)	228 (7.8)	84 (11.6)	55 (7.5)	51 (7.0)	38 (5.2)	<0.001
Greatest weight (kg)	81.65 [71.21, 96.16]	83.91 [74.16, 97.30]	82.55 [70.76, 95.71]	81.65 [71.21, 95.25]	81.65 [68.95, 96.16]	0.022
Age at greatest weight (years)	41.00 [30.00, 56.00]	50.00 [36.00, 63.00]	43.00 [31.00, 58.00]	39.00 [29.00, 53.00]	35.00 [28.00, 48.00]	<0.001
Least weight (kg)	58.97 [52.16, 68.04]	58.97 [52.16, 68.04]	58.06 [51.71, 68.04]	58.97 [52.16, 68.04]	58.97 [51.26, 68.04]	0.685
Age at least weight (years)	18.00 [18.00, 23.00]	18.00 [18.00, 25.00]	18.00 [18.00, 23.00]	19.00 [18.00, 23.00]	18.00 [18.00, 22.00]	0.237

PIR, Poverty Income Ratio; CVD, Cardiovascular Disease; COPD, Chronic Obstructive Pulmonary Disease.

Non-normally distributed continuous variables are presented as median (Q1, Q3). Categorical variables are presented as frequency (n) and proportion (%).The P value

was calculated with the Kruskal-Wallis test in non-normally distributed continuous variables and the chi-square test in categorical variables.

**Figure 1:**
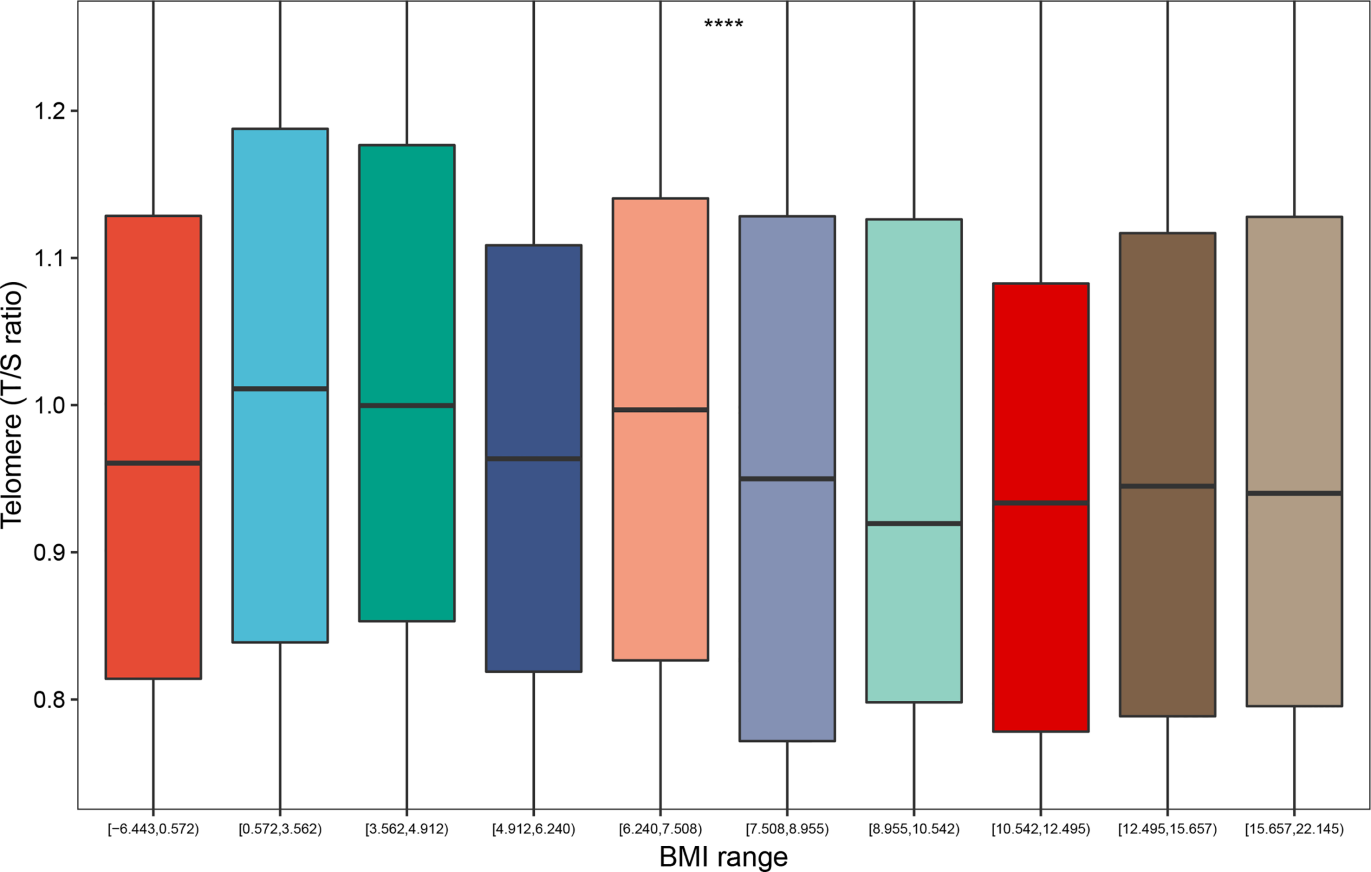
Distribution of telomere length according to the BMI range (deciles). ****: P < 0.0001. BMI range: The difference value between the maximum and minimum of BMI.

As we demonstrated in the boxplot, the distribution of telomere length differed significantly for participants with different BMI range (P < 0.0001); and the BMI range between 0.572 and 3.562 had the largest telomere length (**Figure 1**). This led us to explore the association between weight range and telomere length.

### 3.2 Weight range and telomere length

Presented in **Table 2** were the results of univariate and multivariate linear regression regarding the association between weight range and telomere length. Model 1 was univariate and unadjusted for covariates. BMI_max_, BMI range, and weight range were negatively associated with telomere length. However, annual rate of BMI/weight range exhibited a positive association with telomere length. There was no significant association between telomere length and BMImin. In the multivariate linear regression, indicators of weight range were respectively brought into the Model 2-4. After the potential confounders were adjusted for, the inverse associations persisted in BMI_max_ [β=-0.003, 95% confidence interval (CI): -0.004 to -0.001, P<0.001], BMI range (β=-0.002, 95% CI: -0.003 to -0.001, P=0.003), and weight range (β=-0.001, 95% CI: -0.001 to 0.000, P=0.001). Furthermore, annual rate of BMI range (β=-0.026, 95% CI: -0.046 to -0.007, P=0.009) and weight range (β=-0.010, 95% CI: -0.017 to -0.003, P=0.007) also presented negative associations with telomere length, after adjusting for covariates in Model 2-4. The association between BMI_min_ (β=-0.002, 95% CI: -0.004 to 0.001, P=0.237) and telomere length still could not reach statistical significance in multivariate linear regression model.

**Table 2:** Association of telomere length (T/S ratio) with the weight range.

	Model 1		Model 2		Model 3		Model 4	
Items	β (95% CI)	*P*-value	β (95% CI)	*P*-value	β (95% CI)	*P*-value	β (95% CI)	*P*-value
BMI_max_	-0.004 (-0.005,-0.002)	<0.0001	-0.003 (-0.004,-0.001)	<0.001	-0.003 (-0.004,-0.001)	<0.001	-0.003 (-0.004,-0.001)	<0.001
BMI_min_	-0.001 (-0.004,0.002)	0.382	-0.001 (-0.004, 0.001)	0.275	-0.001 (-0.004, 0.001)	0.274	-0.002 (-0.004, 0.001)	0.237
BMI range	-0.003 (-0.004,-0.001)	<0.001	-0.002 (-0.003,-0.001)	0.004	-0.002 (-0.003,-0.001)	0.004	-0.002 (-0.003,-0.001)	0.003
Weight range	-0.001 (-0.001,0.000)	<0.001	-0.001 (-0.001, 0.000)	0.002	-0.001 (-0.001, 0.000)	0.002	-0.001 (-0.001, 0.000)	0.001
Annual rate of BMI range	0.048 (0.029,0.068)	<0.0001	-0.026 (-0.046,-0.007)	0.008	-0.027 (-0.046,-0.007)	0.007	-0.026 (-0.046,-0.007)	0.009
Annual rate of weight range	0.018 (0.011,0.025)	<0.0001	-0.010 (-0.017,-0.003)	0.006	-0.010 (-0.017,-0.003)	0.005	-0.010 (-0.017,-0.003)	0.007

Model 1: No covariates were adjusted.

Model 2: Adjusted for age, sex, and ethnicity.

Model 3: Adjusted for covariates in Model 2 plus educational level, family PIR, alcohol use, and smoking status.

Model 4: Adjusted for covariates in Model 3 plus medical comorbidities, including diabetes, hypertension, CVD, COPD, and cancer.

CI, Confidence interval; BMI, Body mass index; BMI_max_, the maximum of BMI; BMI_min_, the minimum of BMI; BMI range, the difference value between the maximum and minimum of BMI; Weight range, the difference value between the maximum and minimum of weight; Annual rate of BMI range, calculated by dividing BMI range by time; Annual rate of weight range, calculated by dividing weight range by time; PIR, Poverty Income Ratio; CVD, Cardiovascular Disease; COPD, Chronic Obstructive Pulmonary Disease

**Figure 2:**
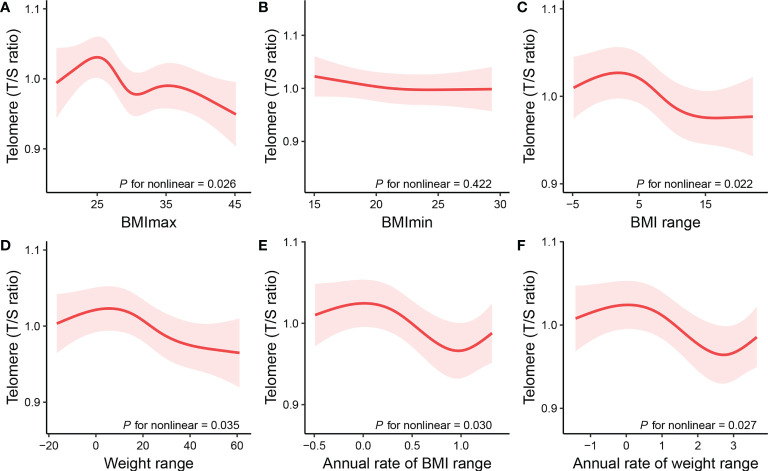
Restricted cubic spline plots of the associations of **(A)** BMI_max_, **(B)** BMI_min_, **(C)** BMI range, **(D)** weight range, **(E)** annual rate of BMI range, and **(F)** annual rate of weight range with telomere length. The associations were adjusted for age, sex, ethnicity, educational level, family PIR, alcohol use, smoking status, and medical comorbidities, including diabetes, hypertension, cardiovascular disease, chronic obstructive pulmonary disease, and cancer. BMI, body Mass Index; BMI_max_, the maximum of BMI; BMI_min_, the minimum of BMI; BMI range, the difference value between the maximum and minimum of BMI; Weight range, the difference value between the maximum and minimum of weight; Annual rate of BMI range, calculated by dividing BMI range by time; Annual rate of weight range, calculated by dividing weight range by time.

To address for nonlinearity, we also used non-parametrically restricted cubic splines analyses to visualize the association of weight range with telomere length. As shown in **Figure 2**, the nonlinear inverse associations were observed in BMI_max_ (P for nonlinear =0.026), BMI range (P for nonlinear =0.022), weight range (P for nonlinear =0.035), annual rate of BMI range (P for nonlinear =0.030), and annual rate of weight range (P for nonlinear =0.027) after adjusting for potential confounders. According to the **Figure 2A**, telomere length reached the maximum around BMI_max_ of 24.9 kg/m², then started to decrease with increasing BMI_max_. The plots in **Figures 2C–F** showed that longer telomere length existed within the lower weight fluctuation. And telomere length remained relatively flat during the changing of BMI_min_ (**Figure 2B**).

### 3.3 Subgroup analysis and sensitivity test

As shown in **Table S2** (**Additional File 1**), two methods of missing values treatment had the same trend. Univariate and multivariate linear regression analyses revealed that inverse association between weight range and telomere length remained but less significant in complete cases analysis.

Based on age, sex, ethnicity, smoking status, alcohol use, subgroup analyses were conducted in Model 1 and Model 4. For BMI_max_, the inverse association with telomere length was still significant in both genders and different age groups. Those Non-Hispanic Blacks, Non-Hispanic Whites, never smokers, moderate and heavy drinkers also showed significant negative associations with telomere length (**Table S3**; **Additional File 1**). The negative association between BMI_min_ and telomere length still could not reach statistical significance in any subgroups (**Table S4**; **Additional File 1**). BMI range correlated negatively with telomere length in male; while the inverse association of weight range with telomere length existed in both genders. For participants who were younger (<60 years), never smoked and drank moderate amounts of alcohol, BMI range and weight range had inverse associations with telomere length (**Tables S5, 6**; **Additional File 1**). The inverse association of annual rate of BMI/weight range with telomere length still statistically significance in male, the younger, former smokers and drinkers (**Tables S7, 8**; **Additional File 1**).

## 4 Discussion

It is a major threat to public health as obesity or overweight is associated with morbidity and mortality related to many diseases, whose prevalence is over 50 percent of total population in the total population, and, on the rise in U.S (24, 25). Emerging studies have proved that obesity which is a multisystem disease (26) is related to telomere length shortening (13, 15, 27). Obesity is commonly diagnosed by BMI; however, BMI range (including the maximum, minimum, range, and annual rate of change), are also anthropometric indicators for clinical evaluation of obesity.

The purpose of this research was to investigate the association of weight range with telomere length. In this retrospective cohort study based on the NHANES database, we found that the baseline BMI of 70 percent of subjects was equal or greater than 25 kg/m². The results of the univariate and multivariate linear regression models indicated that indicators of weight range (BMI_max_, BMI/weight range, annual rate of BMI/weight range) were negatively associated with telomere length among the adults aged 25 to 84 years old. The outcomes of multivariate linear regression were adjusted for the potential confounders (age, sex, ethnicity, educational level, family PIR, alcohol use, smoking status, and medical comorbidities, including diabetes, hypertension, CVD, COPD, and cancer). Non-parametrically restricted cubic splines analyses illustrated indicators of weight range (BMI_max_, BMI/weight range, annual rate of BMI/weight range) all had non-linear inverse associations with telomere length. Telomere length reached the maximum around lower weight fluctuation, then started to decrease with increasing weight range. The sensitivity test result that two methods of missing values treatment have the same trend, reinforcing the robustness of our results. The findings suggested that maintaining normal and stable weight for as long as possible is crucial to maintaining telomere length, which is associated with aging, multimorbidity, and mortality (1, 28).

Classical research for mechanism suggests that obesity-related metabolic dysregulation causes oxidative stress, resulting in shorter telomeres (29). Fat accumulation induces oxidative stress by activating the pro-oxidant enzyme and decreasing the antioxidant enzymes (30). A high concentration of guanines (G) in telomeric region makes it susceptible to oxidative damage because of the formation of 8-dihydro-2’-deoxyguanosine (8-oxodG) at the GGG triplet in telomere sequence; the molecule 8-oxodG associated with several pre-mutagenic alterations such as single-strand brakes, inadequate replication of the telomeric DNA, accelerates telomere shortening (31). The state of low-grade inflammatory is another possible explanation. The increase in BMI triggers the release of inflammatory mediators such as tumor necrosis factor-alpha, interleukin 6, Creative protein, and fibrinogen, negatively affecting telomerase expression (32, 33). Moreover, Broer et al. discovered the obesity with increased leptin concentrations, was associated with inflammation and reduced telomere length (34). The mechanism underlying could also be explained at the genetic level. The fat mass and obesity associated (FTO) gene, clearly identified to be an obesity-related gene (35), affects expression of upstream and downstream flanking genes, such as retinoblastoma-like 2 protein (Rbl2) gene, which influenced genomic hypomethylation in subtelomeric regions, regulated telomere length indirectly (36, 37). Besides, the 2-oxoglutarate dependent dioxygenase catalytic activity of FTO may regulate gene transcription or telomere length regulation directly through nucleic acid demethylation (38).

The study is an important supplement to previous studies, providing novel evidence for health concept of maintaining stable normal weight. Weight and height are commonly used in clinical setting to assess body size. Baseline weight and weight change have been taken into consideration in previous study, confirming that there existed significant associations of weight and weight change with telomere length (15, 39, 40). However, previous studies ignored the indicators of weight range. To assess weight fluctuation, different from the past, we calculate the weight range throughout the lifetime, and the rate and direction (weight gain or loss) were taken into consideration. The study has clearly shown that no matter weight gain or loss, larger weight fluctuation would accelerate telomere length shortening. From this, indicators of weight range are also proved to be of clinical value in body-size assessment. Moreover, the results that weight range were nonlinearly and negatively correlated with telomere length were consistent with the trend of previous studies, suggesting the importance of maintaining stable normal weight, complementing previous research. In the study, some strengths are evident. Considering the impact of missing data on the results, we applied different treatments based on the type of missing data. Meanwhile, sensitivity analyses based on treatments of missing data were carried out. Besides, the statistical analysis included a procedure for dealing with outliers to eliminate the influence on the regression equations. Furthermore, to solve the nonlinearity problem, non-parametrically restricted cubic splines was used to illustrate the associations with telomere length.

There also existed some limitations in our study. Firstly, the observational retrospective cohort study had methodological limitations. Data regarding weight range and time were derived from the recollections of subjects, inevitably carrying the risk of recall bias. Secondly, the indicators of weight range were employed as the only evaluation index because NHANES lacked the data of waist circumference and body fat. However, weight and height were insufficient to capture the full view of obesity. Moreover, the indicators, annual rate of weight and BMI change, did not consider the speed of weight change. There may have been subjects who experienced weight changes over a short period followed by weight maintenance and subjects who changed weight at a slower rate but continuously.

The study of telomeres has contributed to a deeper understanding of human survival, diseases, and aging. Our results of the nonlinearly inverse associations between weight range and telomere length, further support the theory of maintaining stable normal weight and affirm the clinical value of weight range in body-size assessment. Further studies are needed for verification and investigation of causality.

## Data availability statement

The original contributions presented in the study are included in the article/**Supplementary Material**. Further inquiries can be directed to the corresponding authors.

## Ethics statement

The studies involving human participants were reviewed and approved by NCHS Research Ethics Review Board. The patients/participants provided their written informed consent to participate in this study.

## Author contributions

XW and XQ conceived the idea of the study. XW, JW and QQ provided major contributions *via* performing the data analysis, and finishing the manuscript. SG and LZ assisted in the data analysis and confirmed the accuracy. XQ and YL performed critical reviews of the articles. All authors read and approved the final manuscript.
