# Supplementary material for: Association of weight range with telomere length: A retrospective cohort study
Source: Front Endocrinol (Lausanne). 2023 Apr 11;14:1106283. doi: 10.3389/fendo.2023.1106283 (PMC10126401; doi:10.3389/fendo.2023.1106283)
Supplement: Supplementary file 1 [file DataSheet_1.docx]

**On-line Supporting Material**

Supplement Figure 1: Flowchart of data included in this study.


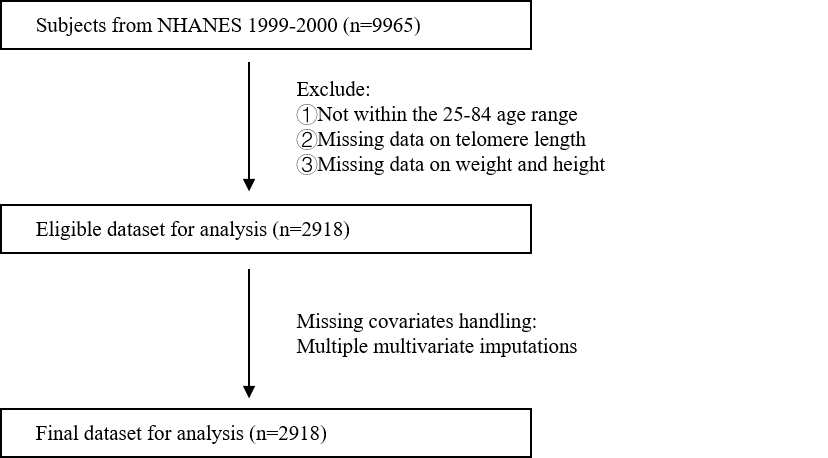


|  | Model1 |  |  | Model2 |  |  | Model3 |  |  | Model4 |  |
| --- | --- | --- | --- | --- | --- | --- | --- | --- | --- | --- | --- |
|  | β (95% CI) | P-value |  | β (95% CI) | P-value |  | β (95% CI) | P-value |  | β (95% CI) | P-value |
| Age at greatest weight | -0.004(-0.004,-0.003) | <0.0001 |  | 0.000(-0.001, 0.001) | 0.612 |  | 0.000(-0.001, 0.001) | 0.704 |  | 0.000(-0.001, 0.001) | 0.876 |

Supplement table 1: The association between age at greatest weight and telomere length

Supplement table 2: Sensitivity analysis: results of linear regression among post-imputation data and complete data

|  | **Post-imputation data** | |  | **Complete data** | |
| --- | --- | --- | --- | --- | --- |
|  | **β (95% CI)** | ***P*-value** |  | **β (95% CI)** | ***P*-value** |
| Participants | 2918 |  |  | 2128 |  |
| BMI_max_ | -0.003(-0.004,-0.001) | <0.001 |  | -0.003(-0.004,-0.001) | 0.002 |
| BMI_min_ | -0.002(-0.004, 0.001) | 0.237 |  | -0.001(-0.004, 0.002) | 0.522 |
| BMI range | -0.002(-0.003,-0.001) | 0.003 |  | -0.002(-0.003, 0.000) | 0.021 |
| Weight range | -0.001(-0.001, 0.000) | 0.001 |  | -0.001(-0.001, 0.000) | 0.017 |
| Annual rate of BMI range | -0.026(-0.046,-0.006) | 0.009 |  | -0.023(-0.046, 0.000) | 0.054 |
| Annual rate of weight range | -0.010(-0.017,-0.003) | 0.007 |  | -0.008(-0.016, 0.000) | 0.053 |

The results of the two methods of missing values treatment had the same trend. The outcomes were adjusted for age, sex, ethnicity, educational level, family PIR, alcohol use, smoking status, and medical comorbidities (diabetes, hypertension, CVD, COPD, and cancer).

CI: Confidence interval; BMI: Body mass index; BMI_max_: the maximum of BMI; BMI_min_ : the minimum of BMI; BMI range: the difference value between the maximum and minimum of BMI; Weight range: the difference value between the maximum and minimum of weight; Annual rate of BMI range: calculated by dividing BMI range by time; Annual rate of weight range: calculated by dividing weight range by time; PIR: Poverty Income Ratio; CVD: Cardiovascular Disease; COPD: Chronic Obstructive Pulmonary Disease

Supplement table 3: Results of subgroup analysis and interaction test (BMI_max_)

|  | **Model1** | |  | **Model4** | |
| --- | --- | --- | --- | --- | --- |
|  | **β (95% CI)** | ***P*-value** |  | **β (95% CI)** | ***P*-value** |
| Overall | -0.004(-0.005,-0.002) | <0.0001 |  | -0.003(-0.004,-0.001) | <0.001 |
| Age |  |  |  |  |  |
| <60 | -0.004(-0.005,-0.002) | <0.0001 |  | -0.003(-0.005,-0.001) | 0.002 |
| ≥60 | -0.002(-0.004,0.001) | 0.224 |  | -0.003(-0.005, 0.000) | 0.037 |
| Sex |  |  |  |  |  |
| Male | -0.004(-0.007,-0.002) | <0.001 |  | -0.004(-0.006,-0.001) | 0.003 |
| Female | -0.003(-0.005,-0.001) | <0.001 |  | -0.003(-0.005,-0.001) | 0.007 |
| Ethnicity |  |  |  |  |  |
| Non-Hispanic white | -0.004(-0.007,-0.002) | <0.0001 |  | -0.003(-0.005,-0.001) | 0.002 |
| Mexican American | -0.002(-0.006,0.001) | 0.116 |  | 0.000(-0.003, 0.004) | 0.794 |
| Non-Hispanic black | -0.005(-0.008,-0.001) | 0.010 |  | -0.005(-0.008,-0.001) | 0.013 |
| Other | -0.005(-0.010,0.000) | 0.060 |  | -0.005(-0.011, 0.001) | 0.084 |
| Smoking |  |  |  |  |  |
| Never | -0.005(-0.007,-0.003) | <0.0001 |  | -0.004(-0.006,-0.001) | <0.001 |
| Former | -0.001(-0.004,0.002) | 0.443 |  | -0.001(-0.004, 0.001) | 0.337 |
| Now | -0.002(-0.006,0.001) | 0.116 |  | -0.003(-0.006, 0.000) | 0.076 |
| Alcohol Use |  |  |  |  |  |
| Never | -0.004(-0.008,0.000) | 0.059 |  | -0.004(-0.008, 0.000) | 0.061 |
| Former | 0.000(-0.003,0.003) | 0.910 |  | -0.001(-0.004, 0.002) | 0.589 |
| Mild | -0.004(-0.007,-0.001) | 0.008 |  | -0.003(-0.005, 0.000) | 0.054 |
| Moderate | -0.006(-0.010,-0.002) | 0.005 |  | -0.006(-0.011,-0.002) | 0.004 |
| Heavy | -0.004(-0.008,-0.001) | 0.018 |  | -0.004(-0.007, 0.000) | 0.049 |

Model 1: no covariates were adjusted.

Model 4: adjusted for age, sex, ethnicity, educational level, family PIR, alcohol use, smoking status, and medical comorbidities, including diabetes, hypertension, cardiovascular disease, chronic obstructive pulmonary disease, and cancer.

CI: Confidence interval; BMI: Body mass index; BMI_max_: the maximum of BMI.

Supplement table 4: Results of subgroup analysis and interaction test (BMI_min_)

|  | **Model1** | |  | **Model4** | |
| --- | --- | --- | --- | --- | --- |
|  | **β (95% CI)** | ***P*-value** |  | **β (95% CI)** | ***P*-value** |
| Overall | -0.001(-0.004,0.002) | 0.382 |  | -0.002(-0.004, 0.001) | 0.237 |
| Age |  |  |  |  |  |
| <60 | -0.001(-0.004,0.003) | 0.626 |  | -0.002(-0.005, 0.001) | 0.250 |
| ≥60 | -0.002(-0.006,0.002) | 0.383 |  | 0.000(-0.005, 0.004) | 0.905 |
| Sex |  |  |  |  |  |
| Male | 0.002(-0.003,0.006) | 0.454 |  | 0.000(-0.004, 0.004) | 0.922 |
| Female | -0.002(-0.006,0.002) | 0.291 |  | -0.003(-0.007, 0.001) | 0.115 |
| Ethnicity |  |  |  |  |  |
| Non-Hispanic white | 0.000(-0.004,0.004) | 0.940 |  | -0.001(-0.005, 0.003) | 0.513 |
| Mexican American | -0.003(-0.009,0.002) | 0.214 |  | -0.001(-0.007, 0.004) | 0.572 |
| Non-Hispanic black | -0.003(-0.010,0.004) | 0.393 |  | -0.004(-0.011, 0.002) | 0.198 |
| Other | 0.004(-0.006,0.014) | 0.447 |  | 0.002(-0.008, 0.013) | 0.680 |
| Smoking |  |  |  |  |  |
| Never | -0.002(-0.006,0.002) | 0.284 |  | -0.003(-0.007, 0.001) | 0.150 |
| Former | 0.001(-0.004,0.007) | 0.573 |  | 0.002(-0.003, 0.007) | 0.386 |
| Now | -0.001(-0.007,0.004) | 0.616 |  | -0.002(-0.008, 0.003) | 0.417 |
| Alcohol Use |  |  |  |  |  |
| Never | -0.004(-0.012,0.004) | 0.380 |  | -0.004(-0.011, 0.004) | 0.376 |
| Former | 0.000(-0.005,0.006) | 0.992 |  | -0.001(-0.006, 0.005) | 0.849 |
| Mild | 0.002(-0.003,0.007) | 0.457 |  | 0.002(-0.003, 0.007) | 0.424 |
| Moderate | -0.004(-0.012,0.005) | 0.369 |  | -0.004(-0.013, 0.005) | 0.394 |
| Heavy | -0.003(-0.009,0.003) | 0.320 |  | -0.005(-0.011, 0.001) | 0.080 |

Model 1: no covariates were adjusted.

Model 4: adjusted for age, sex, ethnicity, educational level, family PIR, alcohol use, smoking status, and medical comorbidities, including diabetes, hypertension, cardiovascular disease, chronic obstructive pulmonary disease, and cancer.

CI: Confidence interval; BMI: Body mass index; BMI_min_ : the minimum of BMI.

Supplement table 5: Results of subgroup analysis and interaction test (BMI range)

|  | **Model1** | | |  | | **Model4** | | |
| --- | --- | --- | --- | --- | --- | --- | --- | --- |
|  | | **β (95% CI)** | ***P*** | |  | | **β (95% CI)** | ***P*** |
| Overall | | -0.003(-0.004,-0.001) | <0.001 | |  | | -0.002(-0.003,-0.001) | 0.003 |
| Age | |  |  | |  | |  |  |
| <60 | | -0.003(-0.005,-0.002) | <0.001 | |  | | -0.002(-0.004, 0.000) | 0.014 |
| ≥60 | | 0.000(-0.002,0.002) | 0.832 | |  | | -0.002(-0.004, 0.000) | 0.099 |
| Sex | |  |  | |  | |  |  |
| Male | | -0.004(-0.006,-0.002) | <0.001 | |  | | -0.003(-0.005,-0.001) | 0.009 |
| Female | | -0.002(-0.004,0.000) | 0.018 | |  | | -0.002(-0.004, 0.000) | 0.067 |
| Ethnicity | |  |  | |  | |  |  |
| Non-Hispanic white | | -0.003(-0.005,-0.002) | <0.001 | |  | | -0.002(-0.003, 0.000) | 0.082 |
| Mexican American | | -0.002(-0.004,0.001) | 0.222 | |  | | -0.001(-0.003, 0.002) | 0.564 |
| Non-Hispanic black | | -0.002(-0.005,0.002) | 0.308 | |  | | -0.002(-0.006, 0.001) | 0.189 |
| Other | | -0.008(-0.013,-0.003) | 0.003 | |  | | -0.007(-0.013,-0.002) | 0.012 |
| Smoking | |  |  | |  | |  |  |
| Never | | -0.004(-0.006,-0.002) | <0.001 | |  | | -0.002(-0.004, 0.000) | 0.026 |
| Former | | -0.001(-0.003,0.002) | 0.456 | |  | | -0.002(-0.004, 0.000) | 0.098 |
| Now | | -0.002(-0.005,0.001) | 0.199 | |  | | -0.002(-0.004, 0.001) | 0.287 |
| Alcohol Use | |  |  | |  | |  |  |
| Never | | -0.002(-0.006,0.002) | 0.378 | |  | | -0.002(-0.007, 0.002) | 0.254 |
| Former | | 0.000(-0.003,0.002) | 0.768 | |  | | -0.001(-0.004, 0.001) | 0.294 |
| Mild | | -0.003(-0.005,0.000) | 0.043 | |  | | -0.002(-0.005, 0.000) | 0.094 |
| Moderate | | -0.005(-0.009,-0.001) | 0.009 | |  | | -0.005(-0.009,-0.002) | 0.006 |
| Heavy | | -0.003(-0.006,0.000) | 0.074 | |  | | -0.001(-0.004, 0.002) | 0.527 |

Model 1: no covariates were adjusted.

Model 4: adjusted for age, sex, ethnicity, educational level, family PIR, alcohol use, smoking status, and medical comorbidities, including diabetes, hypertension, cardiovascular disease, chronic obstructive pulmonary disease, and cancer.

CI: Confidence interval; BMI: Body mass index; BMI range: the difference value between the maximum and minimum of BMI.

Supplement table 6: Results of subgroup analysis and interaction test (Weight range)

|  | **Model1** | |  | **Model4** | |
| --- | --- | --- | --- | --- | --- |
|  | **β (95% CI)** | **P** |  | **β (95% CI)** | **P** |
| Overall | -0.001(-0.001,0.000) | <0.001 |  | -0.001(-0.001, 0.000) | 0.001 |
| Age |  |  |  |  |  |
| <60 | -0.001(-0.002,-0.001) | <0.001 |  | -0.001(-0.002, 0.000) | 0.007 |
| ≥60 | 0.000(-0.001,0.001) | 0.778 |  | -0.001(-0.001, 0.000) | 0.081 |
| Sex |  |  |  |  |  |
| Male | -0.001(-0.002,0.000) | 0.001 |  | -0.001(-0.002, 0.000) | 0.006 |
| Female | -0.001(-0.001,0.000) | 0.059 |  | -0.001(-0.001, 0.000) | 0.048 |
| Ethnicity |  |  |  |  |  |
| Non-Hispanic white | -0.001(-0.002,0.000) | <0.001 |  | -0.001(-0.001, 0.000) | 0.064 |
| Mexican American | -0.001(-0.002,0.000) | 0.151 |  | 0.000(-0.001, 0.001) | 0.446 |
| Non-Hispanic black | -0.001(-0.002,0.001) | 0.260 |  | -0.001(-0.002, 0.000) | 0.164 |
| Other | -0.003(-0.005,-0.001) | 0.004 |  | -0.003(-0.005,-0.001) | 0.011 |
| Smoking |  |  |  |  |  |
| Never | -0.001(-0.002,-0.001) | 0.001 |  | -0.001(-0.002, 0.000) | 0.014 |
| Former | 0.000(-0.001,0.001) | 0.603 |  | -0.001(-0.002, 0.000) | 0.094 |
| Now | -0.001(-0.002,0.000) | 0.153 |  | -0.001(-0.002, 0.000) | 0.195 |
| Alcohol Use |  |  |  |  |  |
| Never | -0.001(-0.002,0.001) | 0.406 |  | -0.001(-0.003, 0.001) | 0.189 |
| Former | 0.000(-0.001,0.001) | 0.815 |  | -0.001(-0.001, 0.000) | 0.270 |
| Mild | -0.001(-0.002,0.000) | 0.065 |  | -0.001(-0.002, 0.000) | 0.051 |
| Moderate | -0.002(-0.003,0.000) | 0.013 |  | -0.002(-0.003,-0.001) | 0.007 |
| Heavy | -0.001(-0.002,0.000) | 0.082 |  | 0.000(-0.002, 0.001) | 0.481 |

Model 1: no covariates were adjusted.

Model 4: adjusted for age, sex, ethnicity, educational level, family PIR, alcohol use, smoking status, and medical comorbidities, including diabetes, hypertension, cardiovascular disease, chronic obstructive pulmonary disease, and cancer.

CI: Confidence interval; BMI: Body mass index; Weight range: the difference value between the maximum and minimum of weight.

Supplement table 7: Results of subgroup analysis and interaction test (Annual rate of BMI range)

|  | **Model1** | |  | | | **Model4** | |
| --- | --- | --- | --- | --- | --- | --- | --- |
|  | **β (95% CI)** | ***P*** | |  | **β (95% CI)** | | ***P*** |
| Overall | 0.048(0.029,0.068) | <0.0001 | |  | -0.026(-0.046,-0.006) | | 0.009 |
| Age |  |  | |  |  | |  |
| <60 | 0.009(-0.014,0.032) | 0.465 | |  | -0.026(-0.049,-0.002) | | 0.034 |
| ≥60 | 0(-0.038,0.039) | 0.983 | |  | -0.02(-0.058, 0.018) | | 0.293 |
| Sex |  |  | |  |  | |  |
| Male | 0.034(0.004,0.063) | 0.027 | |  | -0.037(-0.065,-0.008) | | 0.012 |
| Female | 0.057(0.031,0.083) | <0.0001 | |  | -0.014(-0.042, 0.013) | | 0.307 |
| Ethnicity |  |  | |  |  | |  |
| Non-Hispanic white | 0.046(0.019,0.073) | <0.001 | |  | -0.006(-0.032, 0.019) | | 0.626 |
| Mexican American | 0.037(-0.002,0.076) | 0.064 | |  | -0.039(-0.080, 0.002) | | 0.061 |
| Non-Hispanic black | 0.067(0.015,0.120) | 0.012 | |  | -0.039(-0.099, 0.021) | | 0.201 |
| Other | -0.002(-0.075,0.072) | 0.903 | |  | -0.105(-0.183,-0.028) | | 0.008 |
| Smoking |  |  | |  |  | |  |
| Never | 0.059(0.030,0.088) | <0.0001 | |  | -0.019(-0.048, 0.010) | | 0.205 |
| Former | 0.046(0.008,0.084) | 0.017 | |  | -0.041(-0.078,-0.003) | | 0.035 |
| Now | 0.014(-0.023,0.051) | 0.451 | |  | -0.023(-0.060, 0.014) | | 0.220 |
| Alcohol Use |  |  | |  |  | |  |
| Never | 0.076(0.010,0.141) | 0.024 | |  | -0.048(-0.118, 0.021) | | 0.174 |
| Former | 0.041(-0.001,0.084) | 0.056 | |  | -0.046(-0.089,-0.004) | | 0.034 |
| Mild | 0.051(0.016,0.086) | 0.004 | |  | -0.019(-0.054, 0.016) | | 0.280 |
| Moderate | 0.041(-0.010,0.092) | 0.115 | |  | -0.02(-0.071, 0.031) | | 0.441 |
| Heavy | 0.027(-0.014,0.068) | 0.196 | |  | -0.017(-0.060, 0.025) | | 0.416 |

Model 1: no covariates were adjusted.

Model 4: adjusted for age, sex, ethnicity, educational level, family PIR, alcohol use, smoking status, and medical comorbidities, including diabetes, hypertension, cardiovascular disease, chronic obstructive pulmonary disease, and cancer.

CI: Confidence interval; BMI: Body mass index; Annual rate of BMI range: calculated by dividing BMI range by time.

Supplement table 8: Results of subgroup analysis and interaction test (Annual rate of weight range)

|  | **Model1** | |  | | | **Model4** | |
| --- | --- | --- | --- | --- | --- | --- | --- |
|  | **β (95% CI)** | ***P*** | |  | **β (95% CI)** | | ***P*** |
| Overall | 0.018(0.011,0.025) | <0.0001 | |  | -0.010(-0.017,-0.003) | | 0.007 |
| Age |  |  | |  |  | |  |
| <60 | 0.003(-0.005,0.012) | 0.413 | |  | -0.009(-0.018,-0.001) | | 0.028 |
| ≥60 | -0.001(-0.015,0.013) | 0.915 | |  | -0.008(-0.022, 0.005) | | 0.238 |
| Sex |  |  | |  |  | |  |
| Male | 0.013(0.003,0.023) | 0.014 | |  | -0.014(-0.023,-0.004) | | 0.007 |
| Female | 0.022(0.012,0.032) | <0.0001 | |  | -0.005(-0.015, 0.005) | | 0.316 |
| Ethnicity |  |  | |  |  | |  |
| Non-Hispanic white | 0.017(0.007,0.026) | <0.001 | |  | -0.003(-0.012, 0.006) | | 0.582 |
| Mexican American | 0.012(-0.002,0.026) | 0.091 | |  | -0.015(-0.030, 0.000) | | 0.051 |
| Non-Hispanic black | 0.023(0.004,0.041) | 0.016 | |  | -0.014(-0.035, 0.006) | | 0.175 |
| Other | 0(-0.026,0.027) | 0.971 | |  | -0.038(-0.065,-0.010) | | 0.009 |
| Smoking |  |  | |  |  | |  |
| Never | 0.022(0.012,0.033) | <0.0001 | |  | -0.007(-0.018, 0.003) | | 0.185 |
| Former | 0.017(0.004,0.030) | 0.014 | |  | -0.015(-0.028,-0.001) | | 0.031 |
| Now | 0.005(-0.008,0.018) | 0.469 | |  | -0.009(-0.022, 0.004) | | 0.188 |
| Alcohol Use |  |  | |  |  | |  |
| Never | 0.027(0.003,0.051) | 0.025 | |  | -0.018(-0.043, 0.007) | | 0.169 |
| Former | 0.015(0.000,0.030) | 0.049 | |  | -0.017(-0.032,-0.002) | | 0.032 |
| Mild | 0.019(0.007,0.031) | 0.003 | |  | -0.008(-0.020, 0.005) | | 0.232 |
| Moderate | 0.014(-0.004,0.032) | 0.124 | |  | -0.008(-0.026, 0.010) | | 0.385 |
| Heavy | 0.01(-0.004,0.025) | 0.167 | |  | -0.006(-0.021, 0.009) | | 0.425 |

Model 1: no covariates were adjusted.

Model 4: adjusted for age, sex, ethnicity, educational level, family PIR, alcohol use, smoking status, and medical comorbidities, including diabetes, hypertension, cardiovascular disease, chronic obstructive pulmonary disease, and cancer.

CI: Confidence interval; BMI: Body mass index; Annual rate of BMI range: calculated by dividing weight range by time.

|  |
| --- |
|  |
